# The Iranian National Blood Pressure Measurement Campaign: What Do the Process and Output Evaluation say?

**DOI:** 10.34172/aim.2022.113

**Published:** 2022-11-01

**Authors:** Mohsen Shams, Abbas Pariani, Alireza Raeisi, Mostafa Maleki, Sedigheh Shariatinia, Ahmad Jamalizadeh, Amirhossein Poorkarami, Afshin Ostovar

**Affiliations:** ^1^School of Health, Yasuj University of Medical Sciences, Yasuj, Iran; ^2^Iranian Social Marketing Association, Health Technology Incubation Center, Yasuj University of Medical Sciences, Yasuj, Iran; ^3^Department of Non-Communicable Diseases Management, Deputy of Public Health, Ministry of Health and Medical Education, Tehran, Iran; ^4^School of Medicine, Shiraz University of Medical Sciences, Shiraz, Iran; ^5^Deputy of Public Health, Ministry of Health and Medical Education, Tehran, Iran; ^6^School of Public Health, Tehran University of Medical Sciences, Tehran, Iran; ^7^School of Public Health, Isfahan University of Medical Sciences, Isfahan, Iran; ^8^Health Deputy, Rafsanjan University of Medical Sciences, Rafsanjan, Iran; ^9^Osteoporosis Research Center, Endocrinology and Metabolism Clinical Sciences Institute, Tehran University of Medical Sciences, Tehran, Iran

**Keywords:** Campaign, Hypertension, Output evaluation, Process evaluation

## Abstract

**Background:**

The Iranian National Blood Pressure Measurement Campaign (INBPMC) was conducted all over the country to raise awareness in different groups of people regarding the importance of blood pressure and persuading them to manage their blood pressure. The present research aimed at assessing the process and output of this campaign.

**Methods:**

For process evaluation, 31 universities/faculties were selected. Experts from the Ministry of Health and Medical Education assessed the documentation of the campaign implementation using the designed checklist. The output was assessed by including 8274 people and through a telephone survey using the designed instructions.

**Results:**

The response rate of the telephone interviews was 82.74% (8274 people). It was found that 79% of the selected groups were aware of the INBPMC. Among them, 64% remembered the messages, 99% of the participants who remembered the messages agreed with those messages, and 89% of the participants who agree with the messages went for a measurement of their blood pressure. In the telephone interviews, 30% of the participants stated that they were diagnosed with hypertension, 97% of these participants received the required consultations for subsequent care, and 86% of them went to the health service centers to receive care. The process evaluation of the INBPMC indicated that the universities of medical sciences obtained 97% of the score of the checklist.

**Conclusion:**

The INBPMC was successful and accomplished its objectives.

## Introduction

 Hypertension is one of the most crucial mediating risk factors for non-communicable diseases and is the most important cause of early death around the world.^1,2^ According to the estimations of the World Health Organization (WHO), 1.13 billion people around the world have hypertension, two-thirds of whom live in low- and middle-income countries.^3^ The prevalence of hypertension is expected to increase up to 29% around the world by 2025, which is mainly due to its increase in developing countries.^4^

 The trend of hypertension in Iran has an alarming increase. Evidence shows that the number of hypertensive adults increased in Iran from 1.8 million in 1990 to 13.6 million in 2016. The national age-standardized prevalence of hypertension increased from 8.7% to 28.8% in women and from 8.0% to 24.2% in men from 1990 to 2016.^5^ Moreover, findings of the analysis of 58 primary articles with a sample size of 902 580 showed that the prevalence of hypertension in Iran was 25%. The highest prevalence of hypertension was related to the elderly (42%). The prevalence of hypertension was 25% in women and 24% in men.^6^

 The important issue is that among these people, merely 60% of them were aware of their condition. Half of the patients received medications and the blood pressure was controlled in merely 19% of the patients. In the same report, it was indicated that 32.28% of the population aged 18 years and above were considered within the prehypertension range.^7^

 Despite the knowledge about its risks and the acceptable evidence, most adults are not aware of their hypertension and people with hypertension do not effectively control their blood pressure.^8^ According to estimations, 40%–60% of the Iranians are unaware of their blood pressure status.^9^ Hypertension is of a silent nature and has no symptoms, although being prevalent and leading to serious complications; therefore, most health administrators employ strategies to identify people who are unaware of their blood pressure status.^10^

 The campaign is an effective strategy for encouraging people to have their blood pressure measured and in case they are diagnosed with hypertension, they should start the care program.^11^ Evidence shows that educational interventions have a positive impact on controlling blood pressure and by conducting educational interventions, several changes were made in the lifestyle of people with hypertension.^12^ May Measurement Month (MMM) is the global blood pressure screening campaign, led by the International Society of Hypertension since May 2017.^13,14^ It aims to raise citizens’ awareness of hypertension, collect the scientific evidence needed to help influence global health policy, and make blood pressure screening more widely available around the world through MMM.^13,14^ During May of 2017 and May 2018, over 1.2 and 1.5 million adults, respectively, were screened and in total, over 550 000 adults with untreated or inadequately treated hypertension were identified.^15^

 The Iranian National Blood Pressure Measurement Campaign (INBPMC) was designed to raise awareness among Iranians regarding hypertension, encouraging them to go to the predetermined centers for measurement of their blood pressure, and identifying and providing follow-up care for the people diagnosed with hypertension. The INBPMC commenced from 17/05/2019 (World Hypertension Day) up to 6/07/2019. In this program, all divisions of the Ministry of Health and Medical Education such as management, support, and technical, all universities of medical sciences in Iran, plus all health-related organizations, governmental and non-governmental foundations participated to measure the blood pressure of the Iranians aged 30 years and above, pregnant women, and patients with kidney failure and the required data were collected and recorded. It was the first step in designing and implementing programs for the prevention and management of hypertension in Iranian people. During the implementation of the INBPMC, a collection of educational and informative programs (to inform the target group about hypertension and encourage them to measure their blood pressure and start care actions), and executive actions (to measure the blood pressure of the target group who went to the blood pressure stand, record, and report their data) were carried out. Four months after the implementation of the INBPMC, its process and output were assessed. The objective of output evaluation was to determine the awareness rate of the selected sample of the Iranian population about the National campaign, determining the rate of the received messages, the medium, and resources for receiving the messages, determining the acceptance rate, determining the action rate based on the campaign messages, and determining the visit rate of the target group to the blood pressure measurement stations. The process was assessed to determine whether or not the educational and communicational programs were conducted according to the way they were designed. In this paper, the process and output evaluation of the INBPMC is reported. The results of this evaluation provide a clear picture of the achievements of the INBPMC, which will be useful for similar future programs.

## Materials and Methods

 A designed checklist was used for process evaluation and designed instruction for phone survey was applied for output evaluation of the INBPMC. The estimated proper time for evaluation of the educational and informative programs of the INBPMC ranges from six weeks to six months after its completion. Therefore, four months after the implementation of the INBPMC, its process and output were assessed.

###  Process Evaluation

 For process evaluation, among all universities of medical sciences in Iran, 31 universities/faculties were selected based on their ranking in various academic regions. These universities/faculties included: Ahvaz, Isfahan, Tehran, Tabriz, Shahrood, Shahr-e Kord, Ardabil, Fasa, Bushehr, Iranshar, Yasuj, Gerash, Jiroft, Ilam, Shiraz, Kerman, Zabol, Maragheh, Birjand, Gonabad, Hormozgan, Neishabur, Khalkhal, Kordestan, Larestan, Golestan, Sirjan, Rafsanjan, Babol, Yazd, and Kermanshah. Process evaluation was carried out by experienced managers and experts of the center for management of the non-communicable diseases of the Ministry of Science and Medical Education. To do so, after the required coordination with the authorities of the selected universities/faculties of the medical sciences of the country, the determined assessors completed the respective checklist by visiting the central office of the INBPMC (Health Department of the University/Faculty). The main basis of the responses was the availability and non-availability of the documentation pertinent to the items of the checklist. Examining the documentation and answers to the checklist were carried out in the presence of the officer-in-charge of the non-communicable diseases department. The items of the checklist are shown in Table 1. For validation of the documentation, the assessors randomly selected a healthcare center and by being present in the environment, assessed the respective documentations. Within two months, the checklist of the evaluation process was completed for all universities/faculties.

**Table 1:** Results of Evaluation of the Process of the National Blood Pressure Control Program at the Universities of Medical Sciences in the Country

**Process Evaluation Axis**	**No. (%)**
The Educational & informative program package of the INBPMC is available at the university of medical sciences.	31 (100)
The Educational & informative program package of the INBPMC was sent to the affiliated counties.	31 (100)
The documentations of intra-organizational coordination in the health sector is available in the INBPMC for the implementation of the educational and informative programs.	31 (100)
The documentations of the inter-organizational coordination with the organizations pertinent to the INBPMC are available for the implementation of the educational and informative programs.	31 (100)
The documents for the implementation of the educational programs for the assessors (based on the educational and informative package sent by the Ministry of Health) are available in the university of medical sciences.	31 (100)
The certificate of participation was issued for all assessors of the educational program.	31 (100)
The documentation of the implementation of the educational and informative programs is available in the centers providing health services (health centers, health bases, centers providing healthcare services, and hospitals affiliated to the university).	30 (98)
The educational and informative programs were conducted in the centers providing health services based on the educational and informative packages sent by the Ministry of Health.	28(90)
The documentation of the implementation of the educational and informative programs is available in the clinics and hospitals covered by other organizations (e.g. Social Security Organization, Armed Forces, private sector).	27 (89)
The documentation of the implementation of the educational and informative programs is available in the workplaces (factories, workshops, and offices).	29 (91)
The documentation of the implementation of the educational and informative programs is available in the centers for gatherings of target groups (mosques, subway stations, airports, train stations).	31 (100)
The educational and informative media (such as pamphlet, poster, clip, audio, and video files for computer and mobile) were produced in the university of medical sciences.	31 (100)
The educational and informative media (such as pamphlet, poster, clip, audio, and video files for computer and mobile) were sent to the affiliated counties.	30 (98)
In the educational and informative media (such as pamphlet, poster, clip, audio, and video files for computer and mobile) produced in the university of medical sciences the logo, motto, and messages of the program are included.	31(100)
In the educational and informative programs broadcasted by the I.R Iran Broadcasting, the logo, motto, and messages of the program are included.	30 (98)
The Gantt chart for the educational and informative programs is available in the university of medical sciences.	31 (100)
The educational and informative programs were conducted in the university of medical sciences based on Gantt.	30 (98)
The affiliated counties submitted the report regarding the educational and informative programs to the university.	30 (98)
The affiliated counties of the university of medical sciences provided a Gantt for their educational and informative programs.	30 (98)
The educational and informative programs were conducted in the affiliated counties of the university of medical sciences based on Gantt.	28 (90)

###  Output Evaluation

 The output was assessed through a telephone survey and using the designed instruction. The sample size was calculated to be 9602 (n = 9602) using the Cochran formula, considering the size of the statistical population at 40 000 000 participants, and 0.05% error; for higher precision, the sample size was determined at 10 000. The sample size included 5000 women and 5000 men to observe the sex ratio of the population. The target group for output evaluation included all Iranians aged 30 years and above. Based on the target population covered by the universities/faculties of medical sciences, the proportion was made for sample size, and the share of each university/faculty of medical sciences and their affiliated counties was determined. The investigating team, consisting of trained women and men, called the phone numbers during various times of the day (10 AM to 10 PM) and introduced themselves. They explained the objective of the phone call and after obtaining the consent of the responder, interviewed them according to the designed instruction and recorded the responses. After data collection of the process and the output, the data were analyzed using the SPSS software.

## Results

 The results of the process evaluation of the INBPMC are provided in Table 1. Accordingly, all items were fully conducted and only the following items were not fully investigated in some of the universities:

 The implementation documentation of the educational and informative programs was not available in the health centers of one university of medical sciences (3.22%). The educational programs in the health centers of three universities of medical sciences (9.66%) were not conducted based on the informative and educational package provided by the Ministry of Health. In four universities of medical sciences (12.8%), the documentation of the implementation of the educational and informative programs was not available in the clinics and hospitals covered by other organizations (e.g. Social Security Organization, Armed forces, private sector). The documentation of the implementation of the educational and informative programs was not available in the workplaces (factories, workshops, and offices) of two universities of medical sciences (6.44%). In one university of medical sciences (3.22%), the produced teaser and video clip was not broadcast by the Broadcasting Corporation of the province. In one university of medical sciences (3.22%), the educational and informative programs were not conducted based on the Gantt chart. The counties affiliated to one university of medical sciences (3.22%) did not have a Gantt chart for the implementation of their educational and informative activities and did not send the report of their programs in this regard to the university. In two universities of medical sciences (6.44%), the educational and informative programs were not implemented based on the Gantt chart of the affiliated countries of the universities. Besides, in one university of medical sciences (3.22%), there was no executive office and most of the meetings did not have minutes of meeting. Furthermore, the meetings were held without the president and deputies in these universities.

 Investigating the media receiving the messages of the INBPMC revealed that the health centers, TV, seeing billboards on the streets, and seeing posters were the major ways through which most of the target group were notified of the INBPMC (Table 2). In response to the media for receiving the messages of the INBPMC, 1174 participants (14.19%) stated that they were informed of the campaign by other resources. Among other resources, 341 participants (29%) were informed by mobile stations.

**Table 2:** Relative Frequency Distribution of Sources of Message Delivery in INBPMC

**Sources**	**No. (%)**
Media	
Health centers	2431 (37)
TV	1638 (25)
Billboards	1171 (18)
Posters	741 (11)
Mosques	559 (9)
Parks	393 (6)
Social networks	341 (5)
SMS	340 (5)
Workplace training session	270 (4)
Brochures and pamphlets	260 (4)
Radio	253 (3)
Internet	144 (2)
Social security clinics	132 (2)
Hospital training session	125 (2)
Newspapers and magazines	79 (1)
Other venues	
Mobile health centers	341 (29)
Family, relatives, friends, and acquaintances	312 (26)
Offices, workplaces, schools, and universities	130 (11)
Public places	96 (8)
Hospitals	69 (6)
Terminals and public transportation	58 (5)
Health personnel	29 (3)
Religious places	17 (2)
Others	17 (2)

**Figure 1:**
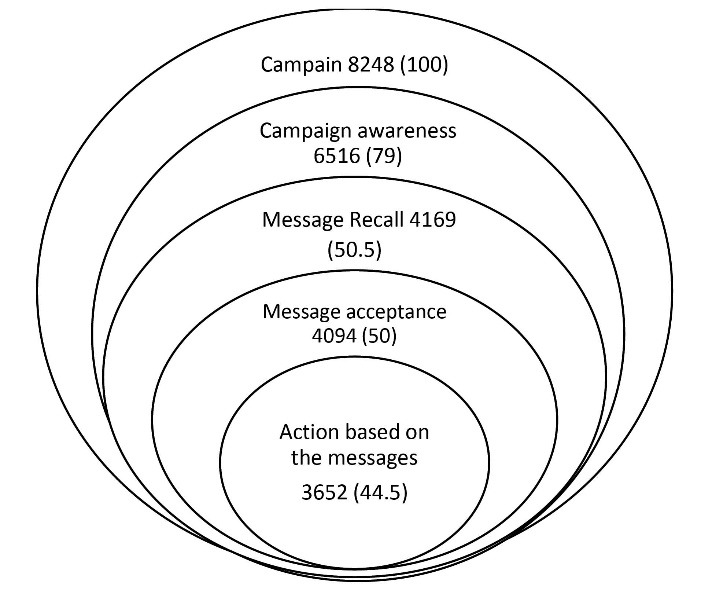
Relative Frequency Distribution of Campaign Awareness, Recall of and Acceptance of Messages and Action Based on Them.

 The sample size for output evaluation of the INBPMC was determined to be 10 000 participants. After a phone call to this number of the selected samples from the Iranian population, a total of 8274 people (82.74%) were willing to participate in the telephone interview. The results of the output evaluation of the INBPMC indicated that 6516 participants from the total number (79%) were aware of the INBPMC. Among them, 4170 (64%) remembered the messages of the INBPMC (50.5% of the interviewed participants), 4049 (99%) of the participants who remembered the messages agreed with the message (50% of the selected target group), and 3652 (89%) of the participants who agreed with the messages of the INBPMC went to the determined centers and bases for blood pressure measurement (44.5% of the selected target group) (Figure 1). Among them, 442 (11%) of the participants who accepted the messages of the INBPMC did not go to the determined blood pressure measurement centers. Their reasons were as follows: measured/controlled in the house, busy schedule and lack of enough time, did not need/seem necessary, under previous care, etc. Furthermore, it was revealed in the telephone interviews that 1072 (30%) of the participants who went for measurement of their blood pressure were diagnosed with hypertension; 1037 (97%) of these people stated that they have received the required instructions and consultations for the subsequent care pertinent to hypertension, and 881 (85%) of the people who received the instructions went to the health service centers to receive the subsequent care pertinent to hypertension.

## Discussion

 Various campaigns used a variety of media and resources for conveying their messages to inform more people in their target group. Taking into account the planning and coordination carried out by the INBPMC, their messages were conveyed to the target group using a different and wide range of media. The results of the process evaluation indicated that the educational and informative packages were available in the universities and the counties affiliated with them and approximately 90% of all promotional, educational, and informational activities were carried out to encourage people to go and measure their blood pressure. These activities were carried out perfectly in the healthcare providing centers, in clinics, and hospitals affiliated to other organizations (e.g. Social Security Organization, Armed Forces, private sector), in workplaces, in the centers for gatherings of target groups (mosques, subway stations, airports, train stations). Besides, the results revealed that in more than 90%, educational and informative programs were conducted. Furthermore, the I.R. IRAN Broadcasting covered the news regarding the INBPMC, and the logo, slogan, name, and tailored messages of the campaign were broadcast in 98% of the cases.

 One of the main reasons for the success of the campaign was due to awareness of more than 70% of the target group about the campaign, using the wide range of various media for introducing the campaign and encouraging people to go to various centers for blood pressure measurement. The variety of the media used for conveying the messages of the campaign was considerable in some campaigns. In the 12/8 Awareness campaign, mass media such as radio, television, billboards, posters, and newspapers were used for conveying the messages of the campaign.^16^

 Another important finding of the study was that health centers, mobile tents and bases, healthcare service providers, and health volunteers were the most important sources for receiving the message by the people. It demonstrates that the health sector and healthcare network have a high potential for conducting such interventions and conveying health messages to the people. Besides, television was the second most frequent source for receiving messages due to its high diffusion among people and covering wider areas.

 Analysis of the process evaluation showed that half of the assessed universities obtained the full score. An overview of the items that scored below 100% and whose results were not received by some assessed universities indicated that the main problem of implementation of the INBPMC was lack of sufficient efforts for documenting the actions.

 In telephone interviews, it was revealed that 30% of the INBPMC participants were diagnosed with hypertension. In other words, the prevalence of hypertension was 30% in this research, which is consistent with the results of the studies around the world. For instance, hypertension was 27.7% in the Caligiuri research.^10^ In the MMM campaign 2017 and 2018, approximately 35% and 33.5% of the participants were diagnosed with hypertension.^17,18^ In another investigation with a sample size of 153,996 and an age range of 35 to 70 years, 40.8% of the people were diagnosed with hypertension.^19^ The results of a similar campaign in 2017 in Venezuela indicated that 48.9% of the people had hypertension.^20^ In this regard, the specialists of the World Heart Federation and the Lancet Commission on Hypertension believe that the main strategy to control hypertension around the world is people’s awareness about their blood pressure status and taking the measures to inform people about the blood pressure in different groups.^21,22^ In general, the studies revealed that implementing campaigns and informing the public about controlling blood pressure and the importance of the complications caused by hypertension is an effective strategy for reducing the number of the diseases caused by hypertension.^18,23^

 The results of the campaigns on the larger scales revealed that the more the awareness of the target group regarding holding campaigns, the more the success of the campaign in achieving its goals. The results of the evaluation of the INBPMC were similar to these studies. For instance, in the evaluation of three great National Anti-Smoking campaigns in America, it was found that in the specific target groups who had more access to the campaign and its messages and were better exposed to the campaign, there was a significant reduction in smoking than the specific target groups with less exposure to the campaign and its results.^24,25^ In accordance with the results of the output evaluation of the INBPMC, the results of the Tips from Former Smokers Campaign demonstrated that the more the access and exposure to the advertisements and messages of the campaign, the higher the chance of the target group being encouraged to adopt that behavior.^26^ In the national campaign of Push Play in New Zealand, with increasing awareness of the target group about the messages and the logo of the campaign, the number of people willing to carrying out physical activities improved significantly.^27^

 The data of the present research were collected in a large sample size and from all over the country. The data of evaluation of the output were collected through telephone interviews and there is the possibility that some of the participants may have provided unreal responses.

 In general, the results of the process and output evaluation of the INBPMC indicated the INBPMC is a proper and very successful national example for addressing health problems, and the Ministry of Health can manage the resources and use its maximum potential to continue designing, implementing, and evaluating campaigns similar to the INBPMC.

 In conclusion, in accordance with the findings of the process evaluation of the INBPMC, it can be concluded that the educational and informative programs were carried out properly in 95% of the cases. The quantitative results of the output evaluation of the INBPMC revealed that the target group was aware of the campaign, remembered its messages, and following the receipt and acceptance of the campaign messages, they referred to the designated centers and bases to measure their blood pressure. A similar approach can be used to improve attention and public awareness about other risk factors of non-communicable diseases.
